# Targeting the Gut Microbiome in Prader-Willi Syndrome

**DOI:** 10.3390/jcm10225328

**Published:** 2021-11-16

**Authors:** Marta Ramon-Krauel, Montse Amat-Bou, Mercedes Serrano, Antonio F. Martinez-Monseny, Carles Lerin

**Affiliations:** 1 Institut de Recerca Sant Joan de Déu, 08950 Barcelona, Spain; marta.ramon@sjd.es (M.R.-K.); montserrat.amat@sjd.es (M.A.-B.); mercedes.serrano@sjd.es (M.S.); antoniofederico.martinez@sjd.es (A.F.M.-M.); 2 Endocrinology Department, Hospital Sant Joan de Déu, 08950 Barcelona, Spain; 3 Neurology Department, Hospital Sant Joan de Déu, 08950 Barcelona, Spain; 4 U703 CIBER-ER, Instituto de Salud Carlos III, 08950 Barcelona, Spain; 5 Clinical Genetics, Department of Genetic and Molecular Medicine/IPER, Hospital Sant Joan de Déu, 08950 Barcelona, Spain

**Keywords:** gut microbiota, probiotics, Prader-Willi syndrome, obesity, mental health symptoms

## Abstract

Overwhelming evidence demonstrates an important role of the gut microbiome in the development of a wide range of diseases, including obesity, metabolic disorders, and mental health symptoms. Indeed, interventions targeting the gut microbiome are being actively investigated as a therapeutic strategy to tackle these diseases. Given that obesity and mental health symptoms are both hallmarks of Prader-Willi syndrome, targeting the gut microbiome may be a promising therapeutical strategy. Only a few studies have investigated the gut microbiome in the context of Prader-Willi syndrome and assessed the efficacy of probiotic supplementation as a therapeutic strategy for this disease. Here, we review the knowledge obtained to this date regarding the gut microbiome in individuals with Prader-Willi syndrome. The limited evidence available indicate that probiotic supplementation improves some metabolic and mental health aspects, however further studies are warranted to determine whether targeting the gut microbiome may constitute a safe and efficient strategy to treat individuals with Prader-Willi syndrome.

## 1. Introduction

### 1.1. Prader-Willi Syndrome

Prader-Willi syndrome (PWS) is a rare genetic disorder with an estimated prevalence of 1/10,000 to 1/30,000 births that affects both genders equally [1,2,3]. PWS is characterized by lack of expression of a set of paternal inherited genes located in the chromosome 15q11.2-q13 region. The lack of gene expression mainly occurs by three different mechanisms: deletion (65–75% of cases), maternal uniparental disomy (20–30% of cases), or imprinting defects (1–3% of cases). Regardless of the genetic origin, the syndrome is associated with a characteristic pattern of dysmorphic features and significant cognitive, behavioral, and endocrine abnormalities.

Individuals with PWS show severe hypotonia and feeding difficulties during infancy [4]. Later on, they typically develop hyperphagia which, in combination with low energy requirements, leads to rapid weight gain [4]. Together with the insatiable hunger due to hypothalamic disturbances, aberrant behaviors typically occur in this latter phase, including food-seeking behavior. Thus, subjects with PWS are at a high risk of developing morbid obesity and associated life-threatening complications unless food intake is strictly controlled by their caregivers. Mental health symptoms including anxiety, depression, and autism spectrum disorders-like symptoms are also common in older children with PWS and constitute a major burden for patients and families [4,5,6]. Notably, subjects with maternal disomy are at higher risk for severe mental health alterations than those with deletions [7].

Current treatments involve early physical and cognitive stimulation, growth hormone therapy, healthy eating patterns, and structured exercise programs, which significantly improve health of subjects with PWS [8]. However, short- and long-term health outcomes are still far from satisfactory. Novel promising therapies are under investigation, including oxytocin, carbetocin, or diazoxide among others [8]. Furthermore, recent studies have focused on the gut microbiome as a target for therapeutical approaches.

### 1.2. The Gut Microbiome in Health and Disease

An increasing number of studies demonstrate the importance of the gut microbiome for the host’s health [9,10,11,12]. The gut microbiome consists in trillions of microorganisms that live in the gastrointestinal tract of mammals and perform important functions for the host, including development of the immune system, preventing growth of pathogenic microorganisms, and fermenting unused substrates. Bacteria can also produce vitamins and other metabolites from dietary nutrients, including short-chain fatty acids, *γ*-aminobutyric acid, serotonin, and other neurotransmitters, which may impact host metabolic and physiologic processes via vagus nerve or through different immune–neuroendocrine mechanisms [13]. Microbiota composition is determined by multiple factors, including age, ethnicity, and diet [14,15].

Numerous studies in humans and animal models have shown that the gut microbiome is an important player in the development of a broad range of diseases, including obesity and metabolic disorders [9,10,16]. An increasing number of clinical trials show that probiotic supplementation with certain bacterial species, including *A. muciniphila* and *B. animalis lactis*, can induce beneficial metabolic effects in overweight subjects [17,18,19]. The gut microbiome can also modulate important central nervous system processes, from satiety mechanisms to anxiety and social behavior [11,12,20]. Therefore, targeting the gut microbiome is being actively investigated as a therapeutic strategy not only for obesity but also for mental health symptoms. In this regards, microbial regulation of tryptophan metabolism, specifically the tryptophan-kynurenine pathway, is being extensively investigated as a mediator of the impact of the gut microbiome on mental health [21,22,23,24].

**Figure 1:**
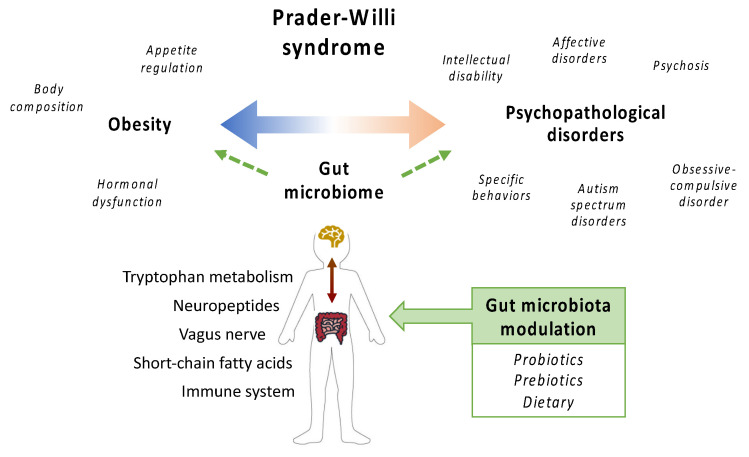
Gut microbiota and Prader-Willi syndrome.

Given the role of intestinal microorganisms in obesity and mental health symptoms, both hallmarks of PWS, the gut microbiome offers a promising target for developing therapeutic strategies (Figure 1). In the present work, we review the knowledge generated on the gut microbiome in the context of PWS as well as the interventions targeting the microbiome in this disease. For this purpose, a literature review up until July 2021 was carried out using PubMed database searches with the following terms: “Prader-Willi syndrome” and “gut/intestinal microbiome/microbiota”. We identified 13 publications that included both observational [25,26,27,28] and interventional studies [29,30,31,32,33], a study protocol [34], and three studies based on secondary analyses of microbiome data from previously published articles [35,36,37]. Publications were classified based on their evidence level in accordance with the Grading of Recommendations Assessment, Development, and Evaluation (GRADE) methodology, considering the lowest evidence level “Expert opinion” (score 4) and the highest evidence level “High quality meta-analysis, systematic reviews of randomized controlled trials (RCTs), or RCTs with a very low risk of a bias” (score 1^++^) [38].

## 2. Characterization of the Prader-Willi Syndrome-Associated Gut Microbiome

Myriad studies have investigated many aspects of the gut microbiome, however only a handful have focused on individuals with PWS (Table 1) [25,26,27,28,29]. While in general these studies suggest that the PWS-associated microbiome has unique characteristics that may contribute to the disease phenotype, the studies are variable in their conclusions. Several factors could explain these discrepancies, including different age-ranges, geographical location, ethnicity, different obesity degrees, and the relatively low number of subjects analyzed.

The first study describing the PWS-associated gut microbiome was published in 2015 by Zhang et al. [29], with 17 children and adolescents with PWS and 21 age-matched subjects with simple obesity. The investigators performed shotgun metagenomic sequencing to analyze the gut microbiome of these subjects and found no major differences in diversity and composition between groups, suggesting that the PWS and simple obesity gut microbiome shared similar structural and functional features. The study also involved a dietary intervention and a longitudinal study of the changes in the gut microbiome, which will be discussed in the next section.

In a different study, Olsson et al. [25] analyzed the fecal microbiota profile of 17 adult subjects with PWS and obesity and 17 matched subjects with simple obesity by sequencing of the V4 region of the bacterial 16S rRNA gene. They observed higher phylogenetic diversity and different overall composition in the PWS group compared to the control group. Specifically, they observed higher abundance of *Akkermansia*, *Desulfovibrio*, and taxa in the Tenericutes phylum and methanogenic Archaea. The authors also observed lower *Dorea* abundance, which have been associated with obesity [39,40,41,42,43]. There were no major differences in the gut microbiome between the genetic subtypes (deletion vs. maternal disomy). Interestingly, the authors also showed that richness and composition of gut microbiota from subjects with PWS was more similar to that from their parents, suggesting that share environment and diet plays an important role in determining gut microbiota composition. The authors also showed that certain microbial taxa prevalent in the PWS microbiota were associated with improved markers of insulin sensitivity independently of body fat mass. Furthermore, in an elegant study transplanting fecal microbiota into germ-free mice, they showed that mice transplanted with microbiota from a donor with PWS had improved insulin tolerance compared to mice transplanted with common obesity microbiota, suggesting a beneficial role of the PWS-associated gut microbiome in glucose metabolism.

Two other studies in children with PWS describing the fecal microbiome were published afterwards. Garcia-Ribera et al. [26] analyzed the microbiome by 16S rRNA gene sequencing (V3-V4 region) in 31 children and adolescents diagnosed with PWS. The authors observed lower phylogenetic diversity and different abundance of several microbial taxa at the genus level in subjects with obesity or overweight compared to those who maintain a normal weight. Three of these genera sustained adjustment for multiple comparisons, with higher *Klebsiella* and lower *Murimonas* and *Alistipes* abundance in the group with obesity. Peng et al. [27] analyzed the gut microbiota by sequencing of the V3-V4 region of the 16S rRNA gene in 25 children with PWS and 25 healthy matched controls. In contrast with the study by Olsson et al. [25], the authors did not observe differences in bacterial diversity or community structure between groups, but identified higher *Prevotella* and lower *Oscillospira* abundance in subjects with PWS. In agreement with the mentioned study [25], they also observed lower *Dorea* abundance in these subjects. Notably, this study also reported the structure of fungal communities (or mycobiome) and showed significant differences in subjects with PWS compared to controls, including higher *Candida* and lower *Saccharomyces* abundance. Furthermore, the authors observed that *Ascomycota* abundance and fungal diversity were inversely and positively associated with hyperphagia symptoms, respectively. In contrast with Garcia-Ribera et al. [26], they found no differences in bacterial phylogenetic diversity between overweight or obese individuals with PWS compared to normal-weight individuals.

More recently, Dahl et al. [28] compared fecal microbiota composition of 25 adults with PWS by 16S sequencing (V3 region) to four other adult cohorts of control healthy individuals with different geographical location and age. They showed that the PWS-associated fecal microbiota profiles differed from those of other adults. In addition, the PWS-associated microbiome profiles have similarities not shared by the other groups, suggesting that PWS has unique characteristics independently of the environment. Adult subjects with PWS exhibited higher abundance of Tenericutes (order RF39), previously identified by Olsson et al. [25], but its relevance to health status is unknown. Furthermore, *Alistipes*, *Parabacteroides*, and *Odoribacter*, as well as *Ruminococcaceae* and *Erysipelotrichaceae* abundance was higher compared to control adults. Some of these bacteria have been inversely associated with cardiometabolic indices and metabolic syndrome [44,45]. Only *Blautia* showed lower abundance in individuals with PWS compared to all other cohorts. *Blautia* abundance has been correlated with higher serum insulin and impaired lipid metabolism [45], suggesting a benefit for low abundance in PWS.

## 3. Targeting the Gut Microbiome as a Therapeutic Approach for Prader-Willi Syndrome

To this date, four randomized controlled clinical studies have evaluated the effects of probiotic supplementation in individuals with PWS using two different bacterial species, namely *B. lactis* and *L. reuteri* without significant adverse events (Table 2) [30,31,32,33]. Additionally, a study implementing a dietary intervention with prebiotics and a fiber-rich diet has also been reported [29].

Zhang et al. [29] conducted a hospitalized intervention with a diet rich in non-digestible carbohydrates in 17 children with PWS in parallel to a group with simple obesity. The intervention improved body weight, metabolic health, and several markers of systemic inflammation. The intervention led to important changes in the gut microbiome, including enrichment in members of the genus *Bifidobacterium*. Subsequent analyses of samples and data from this study unveiled novel information, including the direct contribution of the microbiome to the response to the intervention [29], the potential role of certain strains of *B. pseudocatenulatum* [35], different single-nucleotide polymorphisms (SNPs) in the gut bacteria induced by the dietary intervention [37], and a set of miRNAs regulated by the gut microbiota [36].

Amat-Bou et al. [30] performed a randomized, double-blind, placebo-controlled, crossover study in children and adolescents with PWS to assess the effects of *B. lactis* supplementation (strain BPL1) on obesity and metabolism. Supplementation with BPL1 improved insulin sensitivity and abdominal adiposity, assessed by dual-energy X-ray absorptiometry (DEXA scan). Similar effects of BPL1 on the metabolic status in adult subjects with simple obesity have been previously reported [19]. Furthermore, BPL1 also improved some mental health symptoms, especially in patients with maternal disomy as the genetic cause of the syndrome. No major changes in bacterial communities were observed.

Alyousif et al. [31,34] designed a randomized, double-blind, placebo-controlled, crossover study to assess the effects of *B. lactis* (strain B94) on intestinal wellness and laxation in adult individuals with PWS. They found no changes in microbiota composition and no significant effects of this probiotic on stool frequency or gastrointestinal symptoms. However, they observed differences in stool form during the wash-out period after probiotic suggesting a delayed carry-over effect of the probiotic on intestinal motility [31].

Recently, a larger clinical trial designed to test two different probiotic strains in a cross-over fashion has been completed; results were independently evaluated for each bacterial strain and reported in two different publications as parallel-group clinical studies [32,33]. First, the authors conducted a randomized, double-blind, placebo-controlled 12-week trial to evaluate the efficacy of *B. lactis* (BL-11) on weight, height, and psychological measurements [33]. Sixty-eight subjects with PWS aged 4.2 ± 3.1 years were analyzed. No changes in weight were observed, possibly due to the young mean age of participants and their normal BMI status at the beginning of the study. However, a higher increase in height was observed in the probiotic group, suggesting an interesting effect given the growth hormone deficiency in individuals with PWS. The authors observed probiotic-induced differences in the gut microbiota, including increased *Lactobacillus* and *Prevotella* abundance, which have been previously associated with improved metabolic status [46,47]. While no major changes were found in psychological measurements, the authors reported improvement of the clinician’s view of the patient’s global functioning measured by the Clinical Global Impression Improvement (CGI-I) scale. The second study consisted in a 12-week randomized, double-blind, placebo-controlled trial providing *Limosilactobacillus reuteri* (LR-99) in a cohort of 71 individuals with PWS aged 5.4 ± 4.3 years [32]. Probiotic supplementation significantly reduced BMI and improved social communication, social interaction, and fine motor function. Notably, improvements in social behavior induced by this probiotic had been previously reported in a mouse model of autism spectrum disorder [48]. β-diversity showed a significant separation with probiotic treatment compared to placebo, as also reported in the study with *B. lactis* [33]. Furthermore, the authors also described probiotic-induced changes in the gut microbiota that could contribute to the beneficial effects of the intervention, including lower *Escherichia-Shigella* and higher *Bifidobacterium*, *Lactobacillus*, *Faecalibacterium*, and *Roseburia* abundance.

## 4. Conclusions and Perspectives

Research on the microbiome is rapidly evolving, with a vast number of studies every year generating novel knowledge. Due to the specific characteristics of PWS, these subjects might benefit from probiotic supplementation, especially regarding both metabolic and mental health aspects. The intervention studies to date indicate that probiotic supplementation has the potential to improve adiposity, metabolic parameters, and mental health symptoms in subjects with PWS, with no significant adverse events. While an important first step has been already taken, there are a number of questions that still need to be addressed, especially regarding the potential use of probiotic supplementation as a therapeutic strategy for PWS or to prevent the development of comorbidities in these subjects. There is a lack of data on long-term supplementation effects as all studies were performed for a maximum of 12 weeks. Furthermore, all studies used a single bacterial strain, while a combination of different species (or bacterial consortiums) and/or supplementing with dietary fiber could potentiate the effects. Another important point to consider is the potential convergence or interaction with other therapeutical approaches, and the essential question of whether probiotics might have a synergistic effect with other evidence-based therapies deserves further research. As a chronic condition, early and sustainable interventions may lead to changes in the natural history of the disease, as occurred with the introduction of growth hormone therapy. Moreover, additional studies are needed to further investigate the effects of probiotics on the gut-brain axis and mental health in the PWS context, and to determine whether the genetic cause of the disease plays any role in the response to the intervention. Unfortunately, the underlying mechanism of PWS has not been systematically reported yet, but it is plausible that different clusters of patients show different responses to the same intervention, since their neurological manifestations may be also diverse. In conclusion, targeting the gut microbiome offers a promising therapeutic strategy for individuals with PWS, however further clinical studies are warranted.

## Figures and Tables

**Table 1:** Studies characterizing the gut microbiome in subjects with PWS.

Author, Year	Study Type	Aim	Target Population	Results	GRADE *
Dahl, 2021 [28]	Observational, cross-sectional	PWS adults vs. controls	Mean age 34.9 ± 10.2 years (*n* = 25)	Lower abundance of *Blautia*. Higher RF39 (Tenericutes phyla), *Ruminococcaceae*, *Alistipes*, *Erysipelotrichacaea*, *Parabacteriodes*, *Odoribacter*.	2+
Peng, 2020 [27]	Observational, cross-sectional	PWS children vs. controls	Mean age 6.2 years (range 3–17) (*n* = 25)	Higher *Prevotella* and lower *Oscillospira*. Higher *Candida* and lower *Saccharomyces*. Hyperphagia scores associated with fungal α-diversity.	2++
Garcia-Ribera, 2020 [26]	Observational, cross-sectional	PWS obese vs.PWS normal weight	Mean age 12.0 ± 4.0 years (range 5–18) (*n* = 31)	Lower phylogenetic diversity, lower *Alistipes and Murimonas* abundance, and higher *Klebsiella* abundance in obesity.	2+
Olsson, 2019 [25]	Observational, cross-sectional	PWS obese vs. obese controls	Mean age 29.4 ± 7.8 years (*n* = 17)	Higher phylogenetic diversity in PWS. Higher *Akkermansia*, *Desulfovibrio*, and *Archaea.* Lower *Dorea*.	2++
Zhang, 2015 [29]	Intervention	PWS children with obesity vs. obese controls	Mean age 9.3 years (range 5–16) (*n* = 17)	No major differences in microbiota diversity and composition.	1−

Target population refers to subjects with PWS in the study. * Evidence levels have been classified in accordance with the GRADE methodology [38].

**Table 2:** Intervention studies targeting the gut microbiome in subjects with PWS.

Author, Year	Study Type	Aim	Intervention	Target Population	Time Period	Results	GRADE *
Kong, 2021 [32]	RCT, parallel group	Weight, height, ASQ3, GARS-3	Probiotic *L. reuteri* (LR-99)	Mean age 5.4 ± 4.3 years (*n* = 71)	12 weeks	Decrease in BMI, improvement in social communication and interaction, fine motor function, and total ASQ-3 score	1+
Kong, 2021 [33]	RCT, parallel group	Weight, height, ASQ3, ABC, RRB, SRS-2, CGI-I	Probiotic *B. Lactis* (BL-11)	Mean age 4.2 ± 3.1 years (*n* = 68)	12 weeks	No change in weight, increase in height, improvement in CGI-I	1+
Alyousif, 2020 [31]	RCT, crossover	Stool characteristics and frequency, gastrointestinal symptoms	Probiotic *B. Lactis* (B94)	Mean age 34.9 ± 10.2 years (*n* = 25)	4 weeks	No changes in laxation.	1−
Amat-Bou, 2020 [30]	RCT, crossover	Adiposity, lipid and glucose metabolism, hyperphagia, CBCL	Probiotic *B. Lactis* (BPL1)	Mean age 10.4 ± 5.0 years (*n* = 35)	12 weeks	Decreased abdominal adiposity, improvement in fasting insulin concentration. Modest improvements in CBCL.	1−
Zhang, 2015 [29]	Single group pre-/post-intervention	Microbiota composition, BMI, glucose and lipid homeostasis	Dietary (prebiotics, rich in non-digestive CH)	Mean age 9.3 years (range 5–16) (*n* = 17)	12 weeks	Decrease in weight and inflammation, improved glucose and lipid homeostasis	1−

RCT: randomized clinical trial; BMI: body mass index; ASQ3: Ages & Stages Questionnaires 3rd Edition; GARS-3: Gilliam Autism Rating Scale 3rd Edition; ABC, Aberrant Behavior Checklist; RRB, Restricted Repetitive Behavior scale; SRS-2: Social Responsiveness Scale 2nd Edition; CGI-I: Clinical global impression improvement; CBCL: Child Behavior Checklist. * Evidence levels have been classified in accordance with the GRADE methodology [38].

## Abbreviations

ABC: Aberrant Behavior Checklist; ASQ3: Ages & Stages Questionnaires 3rd Edition; BMI: body mass index; CBCL: Child Behavior Checklist; CGI-I: Clinical global impression improvement; GARS-3: Gilliam Autism Rating Scale 3rd Edition; GRADE, Grading of Recommendations Assessment, Development, and Evaluation; PWS, Prader-Willi syndrome; RCT: randomized clinical trial; RRB, Restricted Repetitive Behavior scale; SRS-2: Social Responsiveness Scale 2nd Edition.
